# Genetic mechanisms in generalized epilepsies

**DOI:** 10.1186/s42494-023-00118-3

**Published:** 2023-03-10

**Authors:** Xiaoqian Wang, Xueyi Rao, Jia Zhang, Jing Gan

**Affiliations:** 1 Department of Pediatrics, West China Second University Hospital, Sichuan University, No. 20, Section Three, South Renmin Road, Chengdu, 610041 China; 2 Key Laboratory of Obstetrics & Gynecologic and Pediatric Diseases and Birth Defects of the Ministry of Education, Sichuan University, No. 20, Section Three, South Renmin Road, Chengdu, 610041 Sichuan China

**Keywords:** Genetic generalized epilepsies, Genetic mechanism, Susceptibility gene, Copy number variations

## Abstract

The genetic generalized epilepsies (GGEs) have been proved to generate from genetic impact by twin studies and family studies. The genetic mechanisms of generalized epilepsies are always updating over time. Although the genetics of GGE is complex, there are always new susceptibility genes coming up as well as copy number variations which can lead to important breakthroughs in exploring the problem. At the same time, the development of ClinGen fades out some of the candidate genes. This means we have to figure out what accounts for a reliable gene for GGE, in another word, which gene has sufficient evidence for GGE. This will improve our understanding of the genetic mechanisms of GGE. In this review, important up-to-date genetic mechanisms of GGE were discussed.

## Introduction

According to the 2022 International League Against Epilepsy (ILAE) classification recommendations, epilepsies with generalized seizure types and generalized spikewave are collectively referred to as "genetic generalized epilepsy" (GGEs). Moreover, there are a few of subgroups classified as childhood absence epilepsy (CAE), juvenile absence epilepsy (JAE), generalized tonic-clonic seizures alone (GTCA) as well as juvenile myoclonic epilepsy (JME) according to the combination of seizure types and the age of onset [1]. There are always overlaps among these GGEs. For example, JME patients could manifest prominent myoclonic seizures otherwise GTCA as well as absence seizures. Moreover, GGE includes individuals with generalized seizure types who do not meet the criteria for a specific syndrome and less common generalized epilepsy syndromes, such as epilepsy with myoclonic absences, epilepsy with eyelid myoclonia, myoclonic epilepsy in infancy and epilepsy with myoclonic-atonic seizures. However, other clinical features including intellectual disability, autism, and abnormal MRI imaging make them excluded from this review.

Basically, one typical GGE could be diagnosed correctly through typical clinical seizures and electrophysiological features followed by successful antiepileptic treatment. However, the hiding etiologies are complicated [2]. Twin studies and family studies have promoted the idiopathic generalized epilepsy to be renamed as genetic generalized epilepsy. Berkovic and collaborators studied epilepsies in twins and showed GGE with a concordance of 76% vs. 33% for monozygotic twins vs. dizygotic twins respectively [3], which was also proved in other studies [4]. This implies genetic factors should be the most important cause of GGE. Besides, family studies suggested siblings had a higher risk of CAE, JAE, and JME than in other epilepsies. These are consistent with genetic diseases [5]. However, the overall rate of epilepsy in siblings of patients with GGE is much lower than expected according to Mendel’s segregation law [6]. Which gives us a clue that the genetic mechanisms underline the GGE are complex and this may be a reason why terminology of idiopathic generalized epilepsy has been preserved so far. This article reviews the articles in the order of summarizing the pathogenesis of common genetic generalized epilepsy and the pathogenic variants of common genes, to the known causative genes, evidence and treatment of specific diseases.

## General genetic considerations

### Pathophysiological mechanisms in genetic generalized epilepsies

GGEs reflect abnormal and highly synchronous neuronal activity in the brain [7]. Where does the devil come from? Genetic pathophysiology in the context of GGEs is so complicated and not uncovered yet. In Chinese philosophy, there is a Yin and Yang theory, which refers to the two opposite and interconnected forces in everything in the world, such as light and dark, fire and water, expanding and contracting. They are relatively connected to each other. The mutual transformation of Yin and Yang is based on the change of Yin and Yang as the premise, that is, the quantitative change leads to qualitative change. As the human body as is concerned, any imbalance of each quality will cause disorders [8]. That may explain the pathophysiological mechanisms in GGEs to some extent, such as the imbalance of depolarization and repolarization, excitation and inhibition. The dysfunction of the network within cortical and subcortical structures are thought to be responsible for GGEs [9, 10]. Within the brain, many factors can interrupt the vulnerable neuronal network which would result in an imbalance of excitation and inhibition, such as ion channelopathies, disturbance of neurotransmitters and receptors. Ion channels dysfunction is the earliest robust pathophysiological mechanisms in epilepsy. Defects in voltage-gated sodium channel could decrease plasma membrane conductance to sodium ions in response to depolarizations and decreased the electrical excitability of gamma-aminobutyric acid (GABA) interneurons which leads to hyperexcitability resulting in epilepsy [11, 12]. Voltage-gated K^+^ channels are important for the regulation of neuronal excitability by M-current, through which neuronal hyperexcitability will be induced and repetitive firing will happen in case of loss of function [12]. Voltage-gated Ca^2+^ channels play a key role in controlling diverse biological processes by regulating Ca^2+^ signaling in excitable cells. Perturbation of the thalamocortical circuit is also proved to be important in generating spike-and-wave seizures [13]. The T-type voltage-gated Ca^2+^ channels serve pace making functions in central neurons and modulate the repetitive firing patterns of the thalamocortical network [14, 15]. Bomben and collaborators reported that early P/Q-type release defect, limited to synapses of a single cell-type within the thalamocortical networks, is sufficient to generate absence epilepsy [16]. As the most important inhibitory neurotransmitter in the central nervous system, GABA can produce an inhibitory postsynaptic potential by activating GABA receptors. It behooves impairment of GABAergic inhibition to increase neuronal excitability resulting in seizures [17, 18]. Hyperpolarization-activated cyclic nucleotide-gated channels (HCN) are a group of pacemakers in the brain. Both increase and decrease in current mediated by HCN are able to contribute to neuronal excitability [19, 20]. Beyond the ion channel reports, there may be other molecular factors contribute to the pathophysiology of GGEs. Glucose transporter 1 (GLUT1) is the exclusive worker to supply glucose to the brain to maintain the physiology of central nervous system (CNS). The defect of this transporter may generate generalized discharges with a phenotype of early-onset absence epilepsy by affecting the vulnerable thalamocortical circuitry [21–24].

### Types of genetic alterations contributing to the GGE

As the development of genetic epilepsy over the time, three main types of genetic alterations contributing to the epilepsies were discovered including single gene variant, copy number variants as well as common variants. Single gene variant for GGE means this variant in a specific gene is sufficient to generate GGE [6]. Phenotypes are due to the contribution of the specific single gene variant, without effect from other genes. It can be grouped as autosomal dominant (AD), autosomal recessive (AR), or X-linked which complies with Mendelian laws. Copy number variations are defined as a chromosomal segment of larger than 1 kb that is present at a variable copy number compared with individuals in the human population, including small deletions or duplications [25], which many genetic architectures of neurological disorders are attributed to with poorly understood complex inheritance, including GGE. Common variants are not necessarily disease-causing, existing in all human populations, some of which could result in complex polygenic diseases. These variants have an accumulative effect on the disease phenotype and will be affected largely by the environment.

As we discussed before, there are four distinct phases in the gene discovery of the generalized epilepsies [26]. During the very beginning of genetic epilepsy, with the studies of large families, *SCN1A*, *SCN1B*, and *GABRG2* were identified as genes for monogenic epilepsies [27–29]. Undoubtedly, which shed a light on the mystery of epilepsy genetic research. That’s where the ion channelopathy epilepsies were established. During the last decade, with the candidate gene approach, only few epilepsy-associated genes were reported, such as *GABRA1*, *SLC2A1*, *CACNA1H* and *EFHC1*, of which the latter two remain debatable [22, 30, 31]. This is the dark time of gene discovery of GGE which implied that the candidate gene approach is not efficient for gene discovery. After entering the era of copy number variation (CNV) analysis and genome-wide association studies (GWASs), microdeletion at 15q13.3, 16p13.11, and 15q11.2 were identified in GGE [32, 33]. Lal et al. have broadened the spectrum of CNV landscape in GGE including microdeletions at 1q21.1, 15q11.2, 15q13.3, 16p11.2, 16p12, 16p13.11 and 22q11.21 [34]. This broadened the types of genetic alterations contributing to GGE. CNVs usually overlap multiple genes and are also seen in the normal population, which makes them be treated as risk factors rather than causative etiologies with complex inheritance. Although there are CNVs related syndromes presenting GGE features along with autism spectrum disorders, intellectual disability, and schizophrenia, which will fail the strict definition of GGE, CNVs carriers with GGE frequently were free of neurodevelopmental disorders. Genome-wide association studies have been applied to investigate common variants contributing to epilepsies, which has achieved an amazing result recently [35]. This tremendous ILAE GWAS published in 2018 involving 15,212 cases and 29,677 controls, which identified that 11 loci were associated with GGE and a third of the genetic basis of GGE was explained by common variants. There were 15 promising candidate genes hiding behind these loci such as *SCN1A*, *SCN2A*, *SCN3A*, *GABRA2*, *KCNN2*, *KCNAB1*, *GRIK1*, *STX1B*, and *PNPO* which can help us understand the causes and novel biological pathways of common epilepsies. Still, there is a lack of sufficient evidence for the definitive risk effect of all the common variants, which should not be applied for counseling purposes and will not be discussed within this review. Nowadays, we have been at the center of the era of massive parallel sequencing, which is dedicated to discovering more and more causative genetic alternations for GGE. Before that, massive exome or genome-sequencing studies projects are needed to better understand the genetic architecture of GGE.

### What accounts for a reliable gene for GGE?

As the rapid development of genetic epilepsy and application of massive parallel sequencing, we are acquiring overwhelming data of genetic studies experimentally and clinically. Here comes another problem: how can we figure out the suspicious gene is the right gene for GGE with clinical validity? What is the sufficient evidence for a reliable gene? No answer may be better than even an unreliable one, which is always the solid belief in genetic counselors. This is definitely true in case we may give the wrong advice on an insufficient gene. If variants of a particular gene have been identified not in the normal population, but recurrently arrested in patients with GGE, this gene should be valid for GGE [36]. There is a robust way to address the issue of defining disease genes: the Clinical Genome Resource (ClinGen) funded by National Institutes of Health (NIH). ClinGen is dedicated to identifying the definitive and causative genes by reviewing genetic and experimental evidence from the scientific literature [37]. It aims at offering an open authoritative knowledge base of genes and variants resources to researchers and clinicians. There are six classification categories for gene-disease validity including: definitive, strong, moderate, limited, disputed, and refuted, by which clinical testing panels and future research studies could be designed. Among the ClinGen Gene Curation working groups, the Epilepsy Gene Curation Expert Panel is one of the earliest approved panels which has developed a framework to standardize the method to determine the clinical validity of an epilepsy candidate gene or variant [38]. This framework is only designed for single gene disease, not for the evaluation of multifactorial conditions. There are two segments within the framework: genetic evidence and experimental evidence. Data of the former one is derived from epilepsy patients which could be grouped into case-level data and case-control data. When evaluating the case-level data, variant evidence and segregation evidence are taken into account. Variant scoring includes autosomal dominant or X-linked disorder and autosomal recessive disorder with different case information considering respectively. The quality of case-control study should be evaluated by variant detection methodology, statistical power, bias, confounding factors and statistical significance. As far as experimental evidence curation is concerned, several factors will be taken into accounts, such as biochemical function, protein interactions, expression, the functional alteration in patient and non-patient derived cells, non-human model, cell culture model as well as rescue by exogenous wild-type gene or gene product. Within the gene curation framework, genetic evidence and experimental evidence could archive a maximum score of 12 points and 6 points separately. It always happened that gene validity could have been proved by enough genetic evidence without experimental evidence, surely which will be reviewed by the Epilepsy Gene Curation Expert Panel. Meanwhile, there are always genes probably innocent from the crime scene. For example, *CACNA1H*, coding for the α-1 subunit of T-type member in voltage-gated Ca^2+^ channels [39], are expressed in cortical neurons and reticular thalamic nucleus [40] which were considered as a candidate gene for genetic generalized epilepsy, especially for childhood absence epilepsy due to its physiology in central nervous system [14, 15]. GABAergic neurons and NMDA-sensitive glutamatergic receptor are found to be related to *CACNA1H* in the genesis of CAE [41, 42]. However, there are no sufficient gene evidence according to the ClinGen criteria. Chen and colleagues found 12 missense pathogenic variants in 14 of the 118 patients in a heterozygous state, which were not identified in any of 230 controls individuals [31]. However, these variants are inherited from unaffected parents, which are not found in Caucasian European patients with childhood absence epilepsy [43]. No de novo variants were reported. Moreover, recent studies have reported that many *CACNA1H* variants are benign polymorphisms with high frequencies [44, 45]. *CACNA1H* might archive some points from experimental evidence [46–48], however, overall, *CACNA1H* as a GGE gene may declare acquittal at present. As far as the human sodium ion channel family is concerned, the researches found that different pathogenic variants in the same gene can lead to different phenotypes. For example, the autism characteristics of *SCN2A* protein truncated carriers (9/13, 69%) are obvious higher than missense pathogenic variant carriers (15/127, 12%, *P* < 0.001), but the timing of seizures was opposite. In addition, early-onset epilepsy occurred in *SCN2A* variants in the effect of gain-of-function (< 3 months), and respond well to sodium channel blockers (SCBs), and in the case of loss-of-function (LoF) variants, patients with late-onset epilepsy and neurodevelopmental disorders (NDDs), are often treatment-resistant. Additionally, in the SCN CNV cohort, we observed that patients with duplications had overt seizures earlier than those with deletions and responded better to SCBs. On the other hand, the article compared the variation of conserved channel positions of different transmembrane channels and found that the functional impact of the two is similar; but their clinical manifestations are different. For example, *SCN1A* and *SCN1B* both have LoF mutations at the same specific point, and the former manifested as degenerative spondylolisthesis, the latter being NDDs/ASD [49]. These results can help us diagnose epilepsy types and identify reliable genes.

## Known genetic etiologies for generalized epilepsies

### Childhood absence seizures: *GABRG2*

**Fig. 1:**
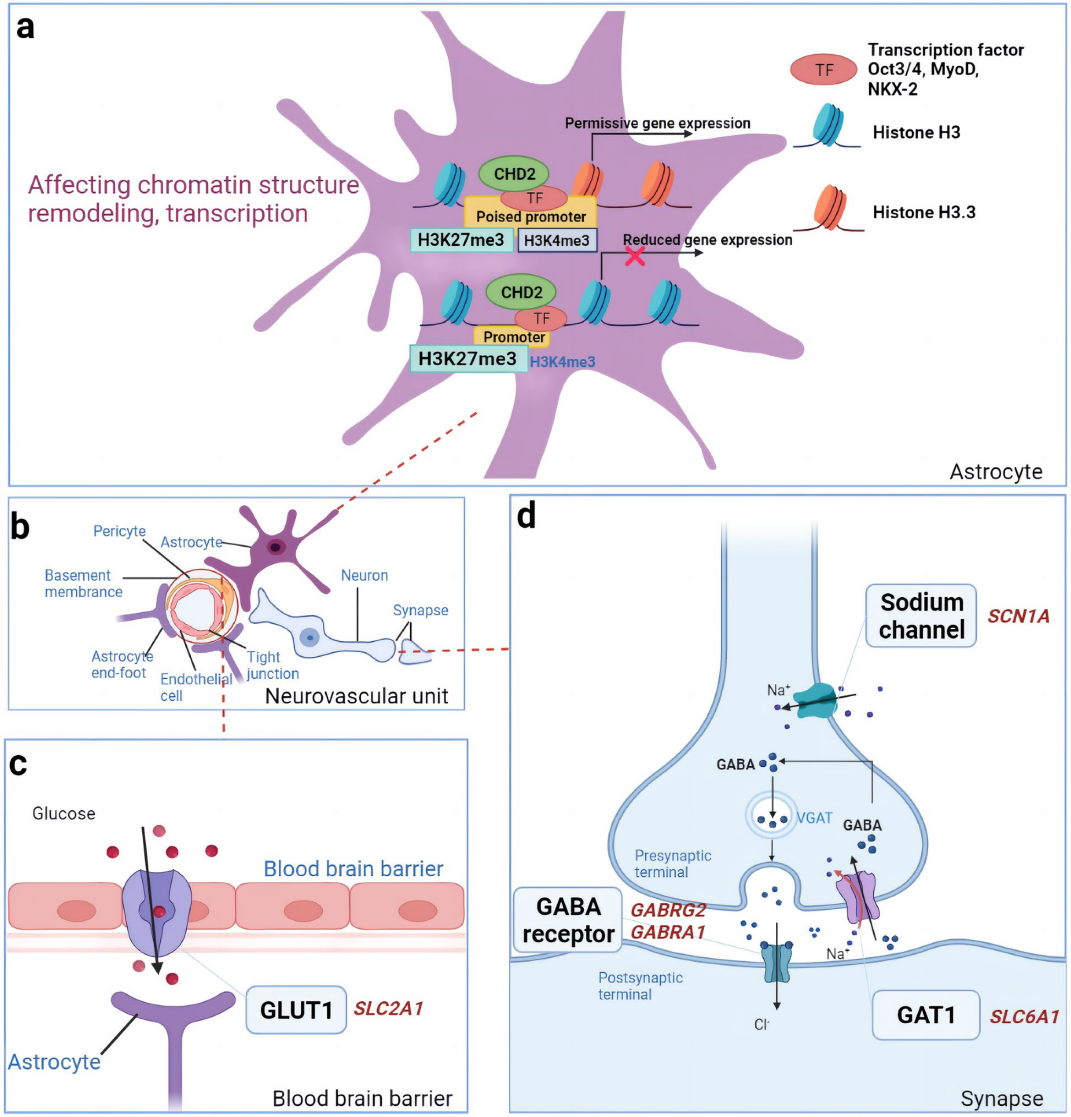
Pathological mechanism of epilepsy caused by pathological variation. **a** The *CHD2* gene encodes a chromatin remodeling protein, and mutations influence the activation of the *H3K4me3* gene, which affects chromosome remodeling. **b** Neurons, astrocytes, pericytes, endothelial cells and other structures consists of the neurovascular unit, and each structure is combined with each other to maintain the homeostasis of the central nervous system. **c ***GABRG2* and *GABRA1* are both the γ-aminobutyric acid (GABA) receptor subunit genes, receptor dysfunction can be caused by the genes pathogenic variation, resulting in increased neuronal excitability and epilepsy. The electrical excitability of GABAergic interneurons, which causes hyperexcitability, could be lowered by defects in voltage-gated sodium channels in response to depolarizations. This the pathological mechanism of *SCN1A* pathogenic variation leading to epilepsy. **d** Insufficient glucose delivery to the brain will result in chronic CNS glucose deprivation due to haploinsufficiency of *SLC2A1* and mild *GLUT1* function loss. The deficiency of this transporter may cause widespread discharges by disrupting the delicate thalamocortical circuitry with a phenotype of early-onset absence epilepsy. *SLC6A1* encodes a GAT1 protein whose receptor reabsorbs GABA

As the most common childhood epilepsy syndrome of GGE, childhood absence seizures are brief, frequent episodes of unconsciousness seizures with possible consequences of impaired attention, mood, and social deficits. Encoding GABA_A_ receptors, *GABRG2* plays an important role in releasing inhibitory neurotransmitters in the brain by promoting chloride ions inflow to hyperpolarize neurons (Fig. 1d). It’s mainly expressed in the brain. Wallace and colleagues found a heterozygous 245G > A pathogenic variant of *GABRG2* cDNA from a 4-generation family study with CAE and febrile seizures (FS) [50]. This pathogenic variant caused p.(R43Q) substitution in the mature GABRG2 protein which contributed to the pathogenesis of both FS and CAE. Given the distinct phenotype and pathophysiology between the two syndromes, this pathogenic variationpathogenic variant has an age-dependent effect. Kananura et al., screened 135 patients with idiopathic absence epilepsy and 154 unrelated and ethnically matched controls for variants of *GABRG2* gene. They reported a point pathogenic variant, IVS6 + 2 T > G, which interrupt the splicing in the mRNA resulting in nonfunctional truncation of the GABA_A_ receptor γ-subunit [51]. These studies suggested *GABRG2* gene pathogenic variant can be a monogenic effect to the pathogenesis of CAE, albeit with rare cases. A pro83-to-ser (P83S) (Table 1) substitution in *GABRG2* was identified in a French Canadian family with idiopathic generalized epilepsy [52]. However, this variant was classified as a variant of unknown significance which needs further investigation. From the experimental evidence aspect, there are also many supporting arguments. By mice models harboring homologous human patients genotypic and phenotypic features, genetic mechanisms of epileptogenesis could be better investigated. Tan and colleagues found a homologous *GABRG2* R43Q pathogenic variation (Table 1) in a knock-in mouse model caused the same phenotype observed in the patients of Wallace’s study [62], which was the earliest syndrome-specific model for genetic epilepsy. The spike-and-wave epileptiform discharges could be blocked by ethosuximide. In heterozygous *Gabrg2*^*+/−*^ knockout mouse models, haploinsufficiency could result in mild absence epilepsy, but not the thermal seizure [63, 64]. Moreover, functional alteration of cortical neurons was showed by impaired inhibitory potential and decreased protein expression. Chiu demonstrated the effect of *GABRG2* R43Q pathogenic variant on the neural network stability and structure by a conditional mouse model and enforced that conclusion that early treatment was needed for overcoming the cascade of developmental issues [65]. All these data together suggested the *GABRG2* R43Q pathogenic variant may be responsible for the absence epilepsy. As treatment is concerned, activators of the GABA_A_ receptors are beneficial with early use. Ethosuximide and valproate are the first-line choice for CAE which are also proved pharmacosensitive in mouse models [62, 66].

**Table 1:** Proposed pathogenic gene, genetic function, variation, and therapeutic plan for reference

GGE	Pathogenic gene	Gene function	Variation discussed in article	Clinical significance	Treatment	Reference
CAE	*GABRG2*	GABA receptors	P83S R43Q	Likely pathogenic Pathogenic	Ethosuximide, Valproate	[51] [52]
JAE	*SLC2A1*	GLUT1 receptor	c.728A > T c.652C > A c.179C > T	Likely pathogenic Conflicting interpretations of pathogenicity conflicting Interpretations of pathogenicity	Ketogenic diet	[53]
JME	*GABRA1*	GABA receptor	A322D Phe104Cys c.-248 + 1G > T	Risk factor Not provided Uncertain significance	Valproate, Vigabatrin	[54] [55]
EEM	*CHD2*	Chromatin remodeling protein	c.3725delA c.4173dupA c.C653T	Pathogenic Pathogenic Likely pathogenic	Valproate, Antisense nucleotides	[56] [57]
EMAtS	*SCN1A*	Voltage-dependent sodium channel	c.3925C > T L433fs*449 c.5104G > T	Conflicting interpretations of pathogenicity Not provided Likely pathogenic	Clobazam, Stiripentol	[58] [59] [60]
	*SLC6A1*	GAT1 receptor	E16X G362R L460R W495X	Not provided	GAT1, Valproic acid, Tiagabine, Vigabatrin	[61]

### Juvenile absence epilepsy: *SLC2A1*

Age at onset of Juvenile absence epilepsy is typically at 8–9 years, with a peak approximately around puberty [1, 2]. Absences are much less frequent than CAEs, with episodes several times per day or less, and loss of consciousness may be less severe [2]. Twin studies have shown that JAE is highly hereditary [67]. In addition, the study conducted by Carla et al. found that the genetic relationship of JAE probands to CAE was roughly the same as expected, but JME was lower than expected. These results support the notion that genetically JAE is relatively distinct from JME and is close to CAE [68]. *SLC2A1*, also known as *GLUT1*, encodes a 492-amino acid protein and contains 10 exons for producing of the major glucose transporter in the brain and erythrocytes [53, 69], which is believed to contribute to rare and severe epileptic encephalopathy (Fig. 1c). However, recent studies have suggested that this gene is also associated with JAE [1]. In vitro functional expression studies in Xenopus oocytes showed that the pathogenic variation resulted in decreased glucose transport or uptake [22]. The main energy source of brain comes from glucose which is exclusively transported by GLUT1 to cross the blood-brain barrier. Haploinsufficiency of *SLC2A1* and mild loss of GLUT1 function and will provide insufficient glucose to the brain, causing a chronic CNS glucose starvation [69, 70]. Todor et al. screened 504 patients with idiopathic generalized epilepsy (IGEs) and identified pathogenic variations of *SLC2A1* in 1% (7/504) of patients. In addition, 3 of them were diagnosed with JAE (c. 728A > T, p. 243E > V; c. 652C > A, p. 218R > S; c. 179C > T, p. 60 T > M) (Table 1), which confirmed the importance of *SLC2A1*. Studies also have shown that these epilepsies cannot be diagnosed with GLUT1 deficiency unless they are accompanied by paroxysmal exertional dyskinesia. The author recommends a low glycemic index diet for relatively mild phenotypes and *SCL2A1* genetic screening if encountering refractory idiopathic epilepsy, with the treatment of the ketogenic diet. Alternative energy bypasses genetic defect as ketone bodies are independent of GLUT1 for membrane transport, which may well treat GLUT1 deficiency caused by *SLC2A1* pathogenic variations [70, 71].

### Juvenile myoclonic epilepsy: *GABRA1*

As one of the first phenotypes to be associated with *GABRA1*, juvenile myoclonic epilepsy is the most common IGE syndrome in adolescents and adults, accounting for approximately 9.3% of all epilepsy types, characterized by myoclonic jerks, GTCA, and absence seizures [1]. A history of CAE is seen in about 5% JME patients. *GABRA1*, along with *GABRG2* harboring a gene cluster in chromosome 5q34 (Fig. 1d) [72], encodes α1-subunit of the postsynaptic GABA-receptors and expresses abundantly in brain [73]. All the known variants with this phenotype have been inherited from an equal or milder affected parent. Patients showed generalized spike-wave discharges without intellectuality affected. Cossette and colleagues reported *GABRA1* with an Ala322Asp variant in a large French Canadian family of JME with autosomal dominant inheritance and segregation for the first time [30]. By comparing with wild-type cells, they found Ala322Asp pathogenic variant cells (Table 1) have lower amplitude of GABA-activated currents, confirming that loss of function of the ligate-gated channel may result in JME [30]. Ding et al. also found that the Ala322Asp pathogenic variant in M3 transmembrane segment result in GABRA1 protein being degraded as a heterozygous loss of function with an altered electrophysiologic property [74]. Moreover, by stopping the wild-type partnering subunits within the endoplasmic reticulum from being transported to the cell surface, Ala322Asp pathogenic variant decreased the expression of GABA_A_ which implied that besides heterozygous loss of function of haploinsufficiency, the Ala322Asp pathogenic variation could result in dominant-negative JME [54, 74]. Bradley and collaborators made further efforts to illuminate that lysosomal degradation and ubiquitin-proteasome system intensively decreased the GABA-receptor expression in HEK293 cells with Ala322Asp pathogenic variant [55]. Johannesen and colleagues reported two JME cases with c.-248 + 1G > T and c.311 T > G (p. F104C) pathogenic variant (Table 1) in *GABRA1* respectively [75]. Through functional study, they found Phe104Cys pathogenic variant led to significant loss-of-function in *Xenopus laevis* oocyte which decreased current amplitudes and GABA sensitivity. The overlap of the phenotype of JME with CAE is also hinted in the mutated *GABRA1* gene. Maljevic reported a de novo pathogenic variant in a German boy with CAE and uncovered a loss of function and haploinsufficiency of the GABA(A) receptor alpha (1)-subunit contributing to the cause of CAE by functional study [76]. Ding and Gallagher studied the dynamics of sensorimotor cortex activation during absence and myoclonic seizures in a mouse model of juvenile myoclonic epilepsy and concluded that both absence and myoclonic seizures shared overlapping networks in sensorimotor cortex [77]. Actually, the phenotypic spectrum of *GABRA1* pathogenic variant is broadly expanded with JAE, generalized epilepsy with febrile seizure (GEFS+) et al. [52, 75]. These finding together demonstrated the contribution of *GABRA1* in GGE. In view of disturbance GABAergic inhibition function by *GABRA1* pathogenic variants, candidate drugs for this defect should aim at increasing the GABA concentration in the synaptic gap. Sodium valproate and vigabatrin are the qualified first-line drugs which have been showed to be able to control seizures of patients with *GABRA1* pathogenic variants [78].

### Generalized tonic-clonic seizures alone

As a common IGE syndrome, Generalized tonic-clonic seizures alone is characterized by sleep deprivation, and 2 h after waking up, usually generalized tonic-clonic seizures occur, and its EEG usually demonstrate generalized spike or multiple spike discharge, with the range from 3 to 5.5 Hz [1]. The age of onset is 5–40 years old, and there is no gender difference [1, 79, 80]. Vorderwülbecke et al. reported that more than 12% (7/57) of first-degree relatives of GTCA patients had a history of seizures [79]. However, the standard diagnostic assessment for GTCA does not include genetic testing. Studies have shown that antiepileptic drugs such as sodium valproate and carbamazepine are effective in treating children with GTCA [80, 81]. Pavlović M et al. reported that the probability of relapse after drug withdrawal in the GTCA group was 80% (8/10) [82]. A cohort study recruiting all IGE patients residing on the Isle of Fuen showed a 0.3% incidence of GTCA and a low risk of GTCA resistance (6.3%) [83]. The 2022 International League Against Epilepsy (ILAE) suggested that doing chromosomal microarray to search for recurrent CNVs if seizures are drug-resistant [1].

### Epilepsy with eyelid myoclonia: *CHD2*

Epilepsy with eyelid myoclonia (EEM), also previously known as Jeavons syndrome, is characterized by a triad of recurrent eyelid myoclonia, either with or without absences, caused on by eye closure and photic stimulation and often generalized tonic-clonic seizures. It is responsible for 1.2 to 2.7% of all epilepsy cases seen, according to several research from epilepsy centers [84–88]. Rhys H. Thomas et al. discovers four cases of EEM of ten patients carrying de novo variants of the *CHD2* (chromo-domain helicase DNA binding protein 2) gene. Each of them had moderate intellectual disability along with photosensitivity, and generalized tonic-clonic seizures [56]. In more than 500 patients with photosensitive epilepsy who performed *CHD2* screenings, Galizia et al. discovered a *CHD2* pathogenic variation or deletion; up to 3/36 (8.3%) of these patients went on to develop a seizure pattern accompanied with eyelid myoclonus (loci c.3725delA, c.4173dupA, c.C653T) (Table 1). These results lead the authors to suggest that *CHD2* is an important contributor to the absence seizures with eyelid myoclonia seizure type [89]. The *CHD2* gene is located on chromosome 15q26.1, contains a total of 39 exons, and is inherited in an autosomal dominant manner. It is thought that *CHD2* belongs to a member of the sucrose nonfermenting (SNF-2) protein family of epigenetic regulators that may contribute to chromatin remodeling and chromatin-based epigenetic modifications (Fig. 1a) [90]. *Z*ebrafish knocked down *CHD2* by using targeted morpholino antisense oligomers exhibited altered locomotor activity and seizure-like epileptiform discharges [91]. A recent finding has confirmed *CHD2* is expressed in the young adult (postnatal day 30 (P30)) mouse brains throughout the brain, and found *CHD2* co-localized in nearly all mature neurons, GABAergic interneurons and oligodendrocytes [57]. These results imply that epileptogenesis and neurodevelopment may be mediated by helicase malfunction. However, our understanding of the function of *CHD2* is still incomplete. In terms of treatment, valproate can inhibit histone deacetylase activity, making histone highly acetylated, and affecting chromosome structure, which may make it an effective drug for diseases related to *CHD2* gene variation [92]. For the precise treatment of diseases caused on by this gene pathogenic variation, thus we need to do numerous studies to identify and confirm. A recent study showed that increased mRNA and protein expression of *CHD2* can be observed in the absence of Chaserr, and loss of *CHD2* function can rescue the Chaserr-deficient phenotype [93]. The results suggest that new treatment methods can be used to make Chaserr loss through antisense nucleotides to increase expression of *CHD2*.

### Epilepsy with myoclonic-atonic seizures: *SCN1A, SLC6A1*

Epilepsy with myoclonic-atonic seizures (EMAtS), also known as Doose syndrome, is defined by myoclonic-atonic seizures in an otherwise normal child who may have a history of febrile or afebrile generalized seizures, affecting 1–2% of children with epilepsy. In spite of the increase in seizures, children with EMAtS exhibit lethargy, fewer social interactions, motor impairments, and greater ataxia consistent with an acute epileptic encephalopathy [58, 94]. The prognosis of EMAtS is uncertain, with outcomes generally unpredictable during the first year of disease. The EEG can be initially normal, and then short pulses and waves, multiple pulses and wave complexes of 2–5 Hz appear with the disease progresses [58].

#### *SCN1A*

*SCN1A* gene encodes the α1 subunit of the sodium ion channel, is located on chromosome 2q24.3, has a total of 26 exons, and is inherited in an autosomal dominant manner (Fig. 1d). Dimova et al. reported a pair of brothers with generalized epilepsy with febrile seizures plus, one with *SCN1A-*mutated myoclonic-atonic epilepsy (c.3925C > T) (Table 1), whose father had a history of one simple febrile seizure in infancy and brother was diagnosed with Dravet syndrome. The patient had an onset of febrile seizures at the age of 3, followed by afebrile generalized tonic-clonic seizures 3 months later, followed by multiple myoclonus and myoclonic-atonic seizures [59]. Ebach et al. screened 20 children with sporadic EMAtS for *SCN1A* gene variation and found that 1 child with EMAS carried *SCN1A* gene variation (L433fs*449) [95] (Table 1). Hinokuma et al. collected 29 patients with Doose syndrome, of which 1 was confirmed by qPCR to have a 588.7 kb de novo deletion involving *SCN1A* at 2q24.3 [60]. A recent study conducted *SCN1A* gene detection on 55 cases of EMAtS, and found a possible pathogenic missense variant (c.5104G > T, p.D1702Y) (Table 1). The patient had a family history of seizures and a history of febrile seizures within 1 year of birth [96]. Not jokingly saying, sodium channel blockers such as lamotrigine and oxcarbazepine are still occasionally prescribed by doctors for *SCN1A* patients. Undoubtedly, the loss-of-function of Na^+^ channels will be exacerbated which will followed by seizure aggravation. Although many disease-target agents have been studying in mouse model or clinically, clobazam and stiripentol are first-line medicine for this issue [97, 98].

#### *SLC6A1*

*SLC6A1* gene is located on chromosome 3 and encodes a voltage-dependent GABA transporter 1 that reabsorbs GABA from the synaptic cleft, which is widely expressed in the mammalian central nervous system, including GABAergic axon terminals astrocytes, oligoden-drocytes and microglia (Fig. 1d) [99, 100]. In a study by Carvill et al., 4% (6/160) probands with MAE were screened for *SLC6A1* pathogenic variations, of which 4 cases were de novo pathogenic variations. In addition, the study presents two independent large-exome sequencing studies reporting two single de novo pathogenic variations in *SLC6A1* in a cohort of individuals with intellectual disability and autism. The study also found that *GAT1*-knockout mice and GAT-1-inhibitor-based mice exhibited spontaneous pulse-wave discharges typical of absence seizures similar to individuals with *SLC6A1* gene variant. These results lead the authors to consider to be an excellent candidate gene for epileptogenesis [101, 102]. Subsequently, A study describing pathogenic *SLC6A1* variants in patients with myoclonic atonic epilepsy (MAE) and intellectual disability (ID) found that 16 of 34 probands with *SLC6A1* pathogenic variation collected were consistent with MAE diagnostic criteria. However, further analysis of the phenotypes of 16 children revealed other epilepsy phenotypes including childhood absence epilepsy, early-onset absence epilepsy, and other unclassified generalized epilepsy disorders [61]. According to a recent study, whole-exome sequencing revealed four de novo pathogenic variations linked to myoclonic atonic epilepsy (loci E16X, G362R, L460R, W495X) (Table 1), and validated all four variants lead to pathogenic variations in *GAT1* in HEK293T, mouse, and human astrocytes, resulting in reduced GABA uptake, by establishing mouse models, radioactive ^3^H-GABA uptake assays and other methods [103]. Therefore, *GAT1* is a potential candidate for therapeutic development targeting *SLC6A1* variant. Well-known antiepileptic drugs such as valproic acid, tiagabine, and vigabatrin improve GABA concentrations [61, 104].

### CNVs: 15q13.3, 15q11.2, 16p13.11

CNVs, which were underestimated before due to the limit of testing, are getting more and more attention because of the novel technologies development such as array comparative genomic hybridization and single nucleotide polymorphism arrays [105]. At the very beginning, CNVs were always associated with neurodevelopmental disorders with complex inheritance, such as intellectual disability, autism, and schizophrenia. Case-control data showed recurrent 15q13.3 microdeletion was identified in about 1% of patients with GGE, including CAE, JME, JAE, GTCS [32, 105, 106]. Lal et al. conducted a genome-wide analysis of 1366 European GGE patients and 5234 genetically matched control patients, and found that microdeletions in the GGE group increased by about 2 times compared with the control group, including a 4.6-fold increase of microdeletions carrying at least one neurodevelopmental disorder-related gene [34]. The article also pointed out that when GGE is combined with other neurodevelopmental disorders, the incidence of recurrent hotspot microdeletions is higher, reflecting its diagnostic value. Additionally, research revealed that one or more rare CNVs were present in 46/517 (8.9%) probands with idiopathic epilepsy, result demonstrated that rare CNVs are important in many subtypes of idiopathic epilepsy, providing important evidence for diagnosis [107]. A recent study also found that in patients with GGE, large repeats were more abundant than large deletions. In addition, deletions of epilepsy genes and epilepsy hotspots were more enrich in GGE compared with controls, such as a deletion on the 15q13.3 recurrent site [108]. One prospective cohort of GGE patients in Bulgaria demonstrated the same frequency of 1% for the 15q13.3 microdeletion [109]. This recurrent deletion contains at least seven genes. Among these genes, *CHRNA7* might be primely responsible for the GGE phenotype, since it plays an important role modulating thalamocortical pathway, encoding a synaptic ion channel protein important for neuronal signaling, which is a susceptibility factor for JME and benign epilepsy in children [110, 111]. Damiano’s study also suggested the complex inheritance of GGE was due to missense pathogenic variants in *CHRNA7* mimicking the way in 15q13.3 microdeletion [112]. These findings point to an important association of microdeletions with affected genes and genetic susceptibility to common GGEs. There are other GGE patients with mild intellectual disability reported to carry a 15q13.3 microdeletion in an autosomal dominant pattern [113]. Significant associations with GGE were also found for the microdeletions at 15q11.2 and 16p13.11 [33]. The odds ratio for the three microdeletions were 68, 4.9, and 7.4 respectively. *CYFIP1* as well as *NIPA2* in 15q11.2 and NDE1 in 16p13.11, like haploinsufficiency of *CHRNA7* in 15q13.3, may be responsible for pathomechanism of GGE [33, 114, 115]. Moreover, deletions at 1q21.1, 16p11.2, and 22q11.2 were found rare but significant in GGE [33, 116]. Surprisingly, most of the GGE patients with these microdeletions harboring genomic hotspots did not have neurodevelopmental disorders comorbidity. Fejgin and collaborators recapitulated myoclonic and absence-like seizures in a 15q13.3 microdeletion mouse model and recorded changes in neuronal excitability [110]. This qualified CNVs as a unique novel effect in genetic architecture of GGE, comparing with monogenetic disease. In a large pediatric cohort, the three CNVs were mainly associated with phenotypes of the spectrum of GGE. However, keep in mind that there were unaffected members also carrying these CNVs and epilepsy patients in the family carrying no CNVs. Moreover, there was lack of segregation pattern or only partial segregation in these family studies. Patients with both GGE and intellectual disability are more vulnerable to carry these CNVs than those with GGE phenotypes alone [117, 118]. Nevertheless, these patients fall outside the strict definition of GGE. All these together, strictly speaking, make the CNVs as risk factors rather than sufficient Mendelian causes for GGE [106, 117]. Interpretation of genetic variation should be combined with clinical phenotype to make diagnosis and treatment of disease. Recently, Jabbari et al. reported 32 in 196 (16%) GGE patients carried rare CNVs in a large WES cohort study. By analyzing protein-protein interaction network, the authors provided a novel genetic bases of GGE by metabolic pathways such as “carbohydrate metabolism”, “small molecule biochemistry” and “cell signaling” which calls for further validations [119].

### Others GGEs: EMA, MEI

Clinically, epilepsy with myoclonic absences is defined by absences characterized by distinct, widespread, rhythmical myoclonias, frequently accompanied by a profound tonic contraction [120]. The average age of onset is 7, with usually range from 3 to 12 years of age. Myoclonic absences (MA) is often combined with other types of epilepsy, particularly with GTCA, and the prognosis will become worse [120, 121]. Myoclonic epilepsy in infancy (MEI) is a rare variety of generalized genetic epilepsies, characterized by the occurrence of brief, generalized myoclonic seizures in children under the age of 3 who are otherwise developmentally normal, without any other seizure types [122]. Moreover, MEI affects boys more than girls and is easily controlled by valproic acid [122–124]. According to the recommendations of the International League Against Epilepsy in 2022, EMA and MEI are often considered polygenic with only specific cases reporting pathogenic variants [1, 84]. The latter has only a few sporadic *TBC1D24* pathogenic variation-related family reports [125]. Therefore, this requires a larger cohort study to find reliable genes and confirm them through whole genome sequencing, modeling and other methods.

## Conclusions

GGEs are very common epilepsies with broad phenotype spectrum resulting from genetic etiologies. Compared with the fast development of genetic epilepsy, there is a relative stagnation in progress of GGEs. The genetic mechanisms underlying GGEs are complicated and there are many clinical and genetical overlaps. CAE patients are always reported with FS and more vulnerable to evolve into JAE or JME. GEFS+ can be accompanied by FS, CAE, JME, MAE et al. There are indeed many other phenotypes of the genes discussed above, such as generalized tonic-clonic, absence, myoclonic, and complex partial seizures in patients with *SLC2A* variants; CAE, Dravet syndrome, as well as patients with just a few seizures in patients with *GABRA1* variants. Further studies are needed to better understand the architecture of GGEs in the future. GWAS and next-generation sequencing (NGS) offer us broad roads to achieve this goal. Recently, a study on associating ultra-rare coding variants with genetic generalized epilepsy through a case-control whole-exome sequencing study demonstrated that ultra-rare coding variants (URVs) in *GABRG2* is an underlying important risk factor for GGE. Compared with sporadic GGE, it is more pronounced in familial GGE about the relevancy of URVs in genes representing the GABAergic pathway [126]. It shows that this technique could help us further understand the genetic heterogeneity and apparently complex inheritance of GGEs. We are currently in a phase of genomic paradigm shifts from candidate gene approach to a hypothesis-free approach, from a data-poor field of science to a big data science. As an unbiased, stringent, hypothesis-free, and robust method, GWAS provided an insight into the common variants of GGE, albeit common variants made limit contribution to GGE and the outcomes of GWAS were sometimes disappointing. Large-scale NGS including exome and genome-sequencing studies will uncover more and more pathogenic genes in GGEs, which requires international collaboration, data sharing and joint publications in the future.

## Data Availability

Availability of data and materials is not applicable in this study.

## Abbreviations

CAE: Childhood absence epilepsy
CNS: Central nervous system
CNVs: Copy number variations
EEM: Epilepsy with eyelid myoclonia
EMAtS: Epilepsy with myoclonic-atonic seizures
GABA: γ-amino-butyric acid
GEFS+: Generalized epilepsy with febrile seizure
GGE: Genetic generalized epilepsy
GLUT1: Glucose transporter 1
GTCA: Generalized tonic-clonic seizures alone
GWAS: Genome-wide study
IGEs: Idiopathic generalized epilepsy
ILAE: International League Against Epilepsy
JAE: Juvenile absence epilepsy
JME: Juvenile myoclonic epilepsy
MA: Myoclonic absences
MEI: Myoclonic epilepsy in infancy
NGS: Next-generation sequencing
URVs: Ultra-rare coding variants

## References

[1] Hirsch E, French J, Scheffer IE, Bogacz A, Alsaadi T, Sperling MR, et al. ILAE definition of the idiopathic generalized epilepsy syndromes: position statement by the ILAE task force on nosology and definitions. Epilepsia. 2022;63(6):1475–99. 35503716 10.1111/epi.17236

[2] Elmali AD, Auvin S, Bast T, Rubboli G, Koutroumanidis M. How to diagnose and classify idiopathic (genetic) generalized epilepsies. Epileptic Disord. 2020;22(4):399–420. 32782228 10.1684/epd.2020.1192

[3] Berkovic SF, Howell RA, Hay DA, Hopper JL. Epilepsies in twins: genetics of the major epilepsy syndromes. Ann Neurol. 1998;43(4):435–45. 9546323 10.1002/ana.410430405

[4] Kjeldsen MJ, Kyvik KO, Christensen K, Friis ML. Genetic and environmental factors in epilepsy: a population-based study of 11 900 Danish twin pairs. Epilepsy Res. 2001;44(2–3):167–78. 11325572 10.1016/s0920-1211(01)00196-6

[5] Peljto AL, Barker-Cummings C, Vasoli VM, Leibson CL, Hauser WA, Buchhalter JR, et al. Familial risk of epilepsy: a population-based study. Brain. 2014;137(3):795–805. 24468822 10.1093/brain/awt368 PMC3927702

[6] Mullen SA, Berkovic SF, Commission IG, Berkovic SF, Lowenstein DH, Kato M, et al. Genetic generalized epilepsies. Epilepsia. 2018;59(6):1148–53. 29741207 10.1111/epi.14042

[7] Fisher RS, Boas WVE, Blume W, Elger C, Genton P, Lee P, et al. Epileptic seizures and epilepsy: definitions proposed by the international league against epilepsy (ILAE) and the International Bureau for Epilepsy (IBE). Epilepsia. 2005;46(4):470–2. 15816939 10.1111/j.0013-9580.2005.66104.x

[8] Li C-L. A brief outline of Chinese medical history with particular reference to acupuncture. Perspect Biol Med. 1974;18(1):132–43. 4612475 10.1353/pbm.1974.0013

[9] Crunelli V, Leresche N. Childhood absence epilepsy: genes, channels, neurons and networks. Nat Rev Neurosci. 2002;3(5):371. 11988776 10.1038/nrn811

[10] Gloor P, Avoli M, Kostopoulos G. Thalamocortical relationships in generalized epilepsy with bilaterally synchronous spike-and-wave discharge. Generalized epilepsy: neurobiological approaches. 1990. p. 190–212.

[11] Mantegazza M, Catterall WA. Voltage-gated Na+ channels and epilepsy. Epilepsia. 2010;51(s5):9–9.

[12] Martin MS, Dutt K, Papale LA, Dubé CM, Dutton SB, de Haan G, et al. Altered function of the SCN1A voltage-gated sodium channel leads to gamma-aminobutyric acid-ergic (GABAergic) interneuron abnormalities. J Biol Chem. 2010;285(13):9823–34. 20100831 10.1074/jbc.M109.078568 PMC2843231

[13] Destexhe A, McCormick DA, Sejnowski TJ. Thalamic and thalamocortical mechanisms underlying 3 Hz spike-and-wave discharges. Prog Brain Res. 1999;121:289–307. 10551033 10.1016/s0079-6123(08)63080-0

[14] Perez-Reyes E. Molecular characterization of T-type calcium channels. Cell Calcium. 2006;40(2):89–96. 16759699 10.1016/j.ceca.2006.04.012

[15] Talavera K, Nilius B. Biophysics and structure–function relationship of T-type Ca2+ channels. Cell Calcium. 2006;40(2):97–114. 16777221 10.1016/j.ceca.2006.04.013

[16] Bomben VC, Aiba I, Qian J, Mark MD, Herlitze S, Noebels JL. Isolated P/Q calcium channel deletion in layer VI corticothalamic neurons generates absence epilepsy. J Neurosci. 2016;36(2):405–18. 26758833 10.1523/JNEUROSCI.2555-15.2016 PMC4710767

[17] Cherubini E. Phasic GABAA-mediated inhibition. Epilepsia. 2010;5(Suppl 51):13.

[18] Dibbens LM, Feng H-J, Richards MC, Harkin LA, Hodgson BL, Scott D, et al. GABRD encoding a protein for extra-or peri-synaptic GABAA receptors is a susceptibility locus for generalized epilepsies. Hum Mol Genet. 2004;13(13):1315–9. 15115768 10.1093/hmg/ddh146

[19] Dyhrfjeld-Johnsen J, Morgan RJ, Földy C, Soltesz I. Upregulated H-current in hyperexcitable CA1 dendrites after febrile seizures. Front Cell Neurosci. 2008;2:2. 18946517 10.3389/neuro.03.002.2008 PMC2525926

[20] Ludwig A, Budde T, Stieber J, Moosmang S, Wahl C, Holthoff K, et al. Absence epilepsy and sinus dysrhythmia in mice lacking the pacemaker channel HCN2. EMBO J. 2003;22(2):216–24. 12514127 10.1093/emboj/cdg032 PMC140107

[21] Mullen SA, Suls A, De Jonghe P, Berkovic SF, Scheffer IE. Absence epilepsies with widely variable onset are a key feature of familial GLUT1 deficiency. Neurology. 2010;75(5):432–40. 20574033 10.1212/WNL.0b013e3181eb58b4

[22] Suls A, Mullen SA, Weber YG, Verhaert K, Ceulemans B, Guerrini R, et al. Early-onset absence epilepsy caused by mutations in the glucose transporter GLUT1. nn Neurol. 2009;66(3):415–9. 10.1002/ana.21724 19798636

[23] Vaudano AE, Olivotto S, Ruggieri A, Gessaroli G, De Giorgis V, Parmeggiani A, et al. Brain correlates of spike and wave discharges in GLUT1 deficiency syndrome. Neuroimage Clin. 2017;13:446–54. 28116237 10.1016/j.nicl.2016.12.026 PMC5233795

[24] Koch H, Weber YG. The glucose transporter type 1 (Glut1) syndromes. Epilepsy Behav. 2019;91:90–3. 30076047 10.1016/j.yebeh.2018.06.010

[25] Mefford HC, Eichler EE. Duplication hotspots, rare genomic disorders, and common disease. Curr Opin Genet Dev. 2009;19(3):196–204. 19477115 10.1016/j.gde.2009.04.003 PMC2746670

[26] Helbig I. Genetic causes of generalized epilepsies. Semin Neurol. 2015;35(3):288–92. 26060908 10.1055/s-0035-1552922

[27] Escayg A, MacDonald BT, Meisler MH, Baulac S, Huberfeld G, An-Gourfinkel I, et al. Mutations of SCN1A, encoding a neuronal sodium channel, in two families with GEFS+2. Nat Genet. 2000;24(4):343–5. 10742094 10.1038/74159

[28] Wallace RH, Wang DW, Singh R, Scheffer IE, George AL Jr, Phillips HA, et al. Febrile seizures and generalized epilepsy associated with a mutation in the Na+−channel beta1 subunit gene SCN1B. Nat Genet. 1998;19(4):366–70. 9697698 10.1038/1252

[29] Wallace RH, Marini C, Petrou S, Harkin LA, Bowser DN, Panchal RG, et al. Mutant GABA(a) receptor gamma2-subunit in childhood absence epilepsy and febrile seizures. Nat Genet. 2001;28(1):49–52. 11326275 10.1038/ng0501-49

[30] Cossette P, Liu L, Brisebois K, Dong H, Lortie A, Vanasse M, et al. Mutation of GABRA1 in an autosomal dominant form of juvenile myoclonic epilepsy. Nat Genet. 2002;31(2):184–9. 11992121 10.1038/ng885

[31] Chen Y, Lu J, Pan H, Zhang Y, Wu H, Xu K, et al. Association between genetic variation of CACNA1H and childhood absence epilepsy. Ann Neurol. 2003;54(2):239–43. 12891677 10.1002/ana.10607

[32] Helbig I, Mefford HC, Sharp AJ, Guipponi M, Fichera M, Franke A, et al. 15q13.3 microdeletions increase risk of idiopathic generalized epilepsy. Nat Genet. 2009;41(2):160–2. 19136953 10.1038/ng.292 PMC3026630

[33] de Kovel CG, Trucks H, Helbig I, Mefford HC, Baker C, Leu C, et al. Recurrent microdeletions at 15q11.2 and 16p13.11 predispose to idiopathic generalized epilepsies. Brain. 2010;133(Pt 1):23–32. 19843651 10.1093/brain/awp262 PMC2801323

[34] Lal D, Ruppert AK, Trucks H, Schulz H, de Kovel CG, Kasteleijn-Nolst Trenité D, et al. Burden analysis of rare microdeletions suggests a strong impact of neurodevelopmental genes in genetic generalised epilepsies. PLoS Genet. 2015;11(5):e1005226. 25950944 10.1371/journal.pgen.1005226 PMC4423931

[35] Consortium TILAE. Genome-wide mega-analysis identifies 16 loci and highlights diverse biological mechanisms in the common epilepsies. Nat Commun. 2018;9(1):5269. 30531953 10.1038/s41467-018-07524-z PMC6288131

[36] Helbig I, Heinzen EL, Mefford HC, Commission ILAEG, Berkovic SF, Lowenstein DH, et al. Genetic literacy series: primer part 2—paradigm shifts in epilepsy genetics. Epilepsia. 2018;59(6):1138–47. 29741288 10.1111/epi.14193

[37] Rehm HL, Berg JS, Brooks LD, Bustamante CD, Evans JP, Landrum MJ, et al. ClinGen—the clinical genome resource. N Engl J Med. 2015;372(23):2235–42. 26014595 10.1056/NEJMsr1406261 PMC4474187

[38] Strande NT, Riggs ER, Buchanan AH, Ceyhan-Birsoy O, DiStefano M, Dwight SS, et al. Evaluating the clinical validity of gene-disease associations: an evidence-based framework developed by the clinical genome resource. Am J Hum Genet. 2017;100(6):895–906. 28552198 10.1016/j.ajhg.2017.04.015 PMC5473734

[39] Catterall W, Perez-Reyes E, Snutch T, Striessnig J, International Union of Pharmacology. XLVIII. Nomenclature and structure-function relationships of voltage-gated calcium channels. Pharmacol Rev. 2005;57:411–25. 16382099 10.1124/pr.57.4.5

[40] Cheong E, Shin H-S. T-type Ca2+ channels in absence epilepsy. Pflugers Arch. 2014;466(4):719–34. 24519464 10.1007/s00424-014-1461-y

[41] Blumenfeld H. Cellular and network mechanisms of spike-wave seizures. Epilepsia. 2005;46(Suppl 9):21–33. 16302873 10.1111/j.1528-1167.2005.00311.x

[42] Wang G, Bochorishvili G, Chen Y, Salvati KA, Zhang P, Dubel SJ, et al. CaV3. 2 calcium channels control NMDA receptor-mediated transmission: a new mechanism for absence epilepsy. Genes Dev. 2015;29(14):1535–51. 26220996 10.1101/gad.260869.115 PMC4526737

[43] Chioza B, Everett K, Aschauer H, Brouwer O, Callenbach P, Covanis A, et al. Evaluation of CACNA1H in European patients with childhood absence epilepsy. Epilepsy Res. 2006;69(2):177–81. 16504478 10.1016/j.eplepsyres.2006.01.009

[44] Becker F, Reid CA, Hallmann K, Tae HS, Phillips AM, Teodorescu G, et al. Functional variants in HCN 4 and CACNA 1H may contribute to genetic generalized epilepsy. Epilepsia open. 2017;2(3):334–42. 29588962 10.1002/epi4.12068 PMC5862120

[45] Lek M, Karczewski KJ, Minikel EV, Samocha KE, Banks E, Fennell T, et al. Analysis of protein-coding genetic variation in 60,706 humans. Nature. 2016;536(7616):285. 27535533 10.1038/nature19057 PMC5018207

[46] Powell KL, Cain SM, Ng C, Sirdesai S, David LS, Kyi M, et al. A Cav3. 2 T-type calcium channel point mutation has splice-variant-specific effects on function and segregates with seizure expression in a polygenic rat model of absence epilepsy. J Neurosci. 2009;29(2):371–80. 19144837 10.1523/JNEUROSCI.5295-08.2009 PMC6664949

[47] Vitko I, Bidaud I, Arias JM, Mezghrani A, Lory P, Perez-Reyes E. The I–II loop controls plasma membrane expression and gating of Cav3. 2 T-type Ca2+ channels: a paradigm for childhood absence epilepsy mutations. J Neurosci. 2007;27(2):322–30. 17215393 10.1523/JNEUROSCI.1817-06.2007 PMC6672065

[48] Cain SM, Snutch TP. T-type calcium channels in burst-firing, network synchrony, and epilepsy. Biochim Biophys Acta. 2013;1828(7):1572–8. 22885138 10.1016/j.bbamem.2012.07.028

[49] Brunklaus A, Du J, Steckler F, Ghanty II, Johannesen KM, Fenger CD, et al. Biological concepts in human sodium channel epilepsies and their relevance in clinical practice. Epilepsia. 2020;61(3):387–99. 32090326 10.1111/epi.16438

[50] Wallace RH, Marini C, Petrou S, Harkin LA, Bowser DN, Panchal RG, et al. Mutant GABA a receptor γ2-subunit in childhood absence epilepsy and febrile seizures. Nat Genet. 2001;28(1):49–52. 11326275 10.1038/ng0501-49

[51] Kananura C, Haug K, Sander T, Runge U, Gu W, Hallmann K, et al. A splice-site mutation in GABRG2 associated with childhood absence epilepsy and febrile convulsions. Arch Neurol. 2002;59(7):1137–41. 12117362 10.1001/archneur.59.7.1137

[52] Lachance-Touchette P, Brown P, Meloche C, Kinirons P, Lapointe L, Lacasse H, et al. Novel α1 and γ2 GABAA receptor subunit mutations in families with idiopathic generalized epilepsy. Eur J Neurosci. 2011;34(2):237–49. 21714819 10.1111/j.1460-9568.2011.07767.x

[53] Baroni MG, Oelbaum RS, Pozzilli P, Stocks J, Li S-R, Fiore V, et al. Polymorphisms at the GLUT1 (HepG2) and GLUT4 (muscle/adipocyte) glucose transporter genes and non-insulin-dependent diabetes mellitus (NIDDM). Hum Genet. 1992;88(5):557–61. 1348045 10.1007/BF00219344

[54] Krampfl K, Maljevic S, Cossette P, Ziegler E, Rouleau GA, Lerche H, et al. Molecular analysis of the A322D mutation in the GABAA receptor α1-subunit causing juvenile myoclonic epilepsy. Eur J Neurosci. 2005;22(1):10–20. 16029191 10.1111/j.1460-9568.2005.04168.x

[55] Bradley CA, Taghibiglou C, Collingridge GL, Wang YT. Mechanisms involved in the reduction of GABAA receptor α1 subunit expression caused by the epilepsy mutation A322D in the trafficking competent receptor. J Biol Chem. 2008;283(32):22043–50. 18534981 10.1074/jbc.M801708200

[56] Thomas RH, Zhang LM, Carvill GL, Archer JS, Heavin SB, Mandelstam SA, et al. CHD2 myoclonic encephalopathy is frequently associated with self-induced seizures. Neurology. 2015;84(9):951–8. 25672921 10.1212/WNL.0000000000001305 PMC4351660

[57] Kim YJ, Khoshkhoo S, Frankowski JC, Zhu B, Abbasi S, Lee S, et al. Chd2 is necessary for neural circuit development and long-term memory. Neuron. 2018;100(5):1180–93.e6. 30344048 10.1016/j.neuron.2018.09.049 PMC6479120

[58] Kelley SA, Kossoff EH. Doose syndrome (myoclonic-astatic epilepsy): 40 years of progress. Dev Med Child Neurol. 2010;52(11):988–93. 20722665 10.1111/j.1469-8749.2010.03744.x

[59] Dimova PS, Yordanova I, Bojinova V, Jordanova A, Kremenski I. Generalized epilepsy with febrile seizures plus: novel SCN1A mutation. Pediatr Neurol. 2010;42(2):137–40. 20117752 10.1016/j.pediatrneurol.2009.09.007

[60] Hinokuma N, Nakashima M, Asai H, Nakamura K, Akaboshi S, Fukuoka M, et al. Clinical and genetic characteristics of patients with Doose syndrome. Epilepsia Open. 2020;5(3):442–50. 32913952 10.1002/epi4.12417 PMC7469791

[61] Johannesen KM, Gardella E, Linnankivi T, Courage C, de Saint MA, Lehesjoki AE, et al. Defining the phenotypic spectrum of SLC6A1 mutations. Epilepsia. 2018;59(2):389–402. 29315614 10.1111/epi.13986 PMC5912688

[62] Tan HO, Reid CA, Single FN, Davies PJ, Chiu C, Murphy S, et al. Reduced cortical inhibition in a mouse model of familial childhood absence epilepsy. Proc Natl Acad Sci U S A. 2007;104(44):17536–41. 17947380 10.1073/pnas.0708440104 PMC2077291

[63] Warner TA, Shen W, Huang X, Liu Z, Macdonald RL, Kang J-Q. Differential molecular and behavioural alterations in mouse models of GABRG2 haploinsufficiency versus dominant negative mutations associated with human epilepsy. Hum Mol Genet. 2016;25(15):3192–207. 27340224 10.1093/hmg/ddw168 PMC5179921

[64] Reid CA, Kim T, Phillips AM, Low J, Berkovic SF, Luscher B, et al. Multiple molecular mechanisms for a single GABAA mutation in epilepsy. Neurology. 2013;80(11):1003–8. 23408872 10.1212/WNL.0b013e3182872867 PMC3653202

[65] Chiu C, Reid CA, Tan HO, Davies PJ, Single FN, Koukoulas I, et al. Developmental impact of a familial GABAA receptor epilepsy mutation. Ann Neurol. 2008;64(3):284–93. 18825662 10.1002/ana.21440 PMC3707613

[66] Kim TH, Reid CA, Petrou S. Oxcarbazepine and its active metabolite,(S)-licarbazepine, exacerbate seizures in a mouse model of genetic generalized epilepsy. Epilepsia. 2015;56(1):e6–9. 25489632 10.1111/epi.12866

[67] Vadlamudi L, Milne RL, Lawrence K, Heron SE, Eckhaus J, Keay D, et al. Genetics of epilepsy: the testimony of twins in the molecular era. Neurology. 2014;83(12):1042–8. 25107880 10.1212/WNL.0000000000000790 PMC4166361

[68] Marini C, Scheffer IE, Crossland KM, Grinton BE, Phillips FL, McMahon JM, et al. Genetic architecture of idiopathic generalized epilepsy: clinical genetic analysis of 55 multiplex families. Epilepsia. 2004;45(5):467–78. 15101828 10.1111/j.0013-9580.2004.46803.x

[69] Wang D, Kranz-Eble P, De Vivo DC. Mutational analysis of GLUT1 (SLC2A1) in Glut-1 deficiency syndrome. Hum Mutat. 2000;16(3):224–31. https://doi.org/10.1002/1098-1004(200009)16:3<224::AID-HUMU5>3.0.CO;2-P . 10980529 10.1002/1098-1004(200009)16:3<224::AID-HUMU5>3.0.CO;2-P

[70] Arsov T, Mullen SA, Rogers S, Phillips AM, Lawrence KM, Damiano JA, et al. Glucose transporter 1 deficiency in the idiopathic generalized epilepsies. Ann Neurol. 2012;72(5):807–15. 23280796 10.1002/ana.23702

[71] Weber YG, Storch A, Wuttke TV, Brockmann K, Kempfle J, Maljevic S, et al. GLUT1 mutations are a cause of paroxysmal exertion-induced dyskinesias and induce hemolytic anemia by a cation leak. J Clin Invest. 2008;118(6):2157–68. 18451999 10.1172/JCI34438 PMC2350432

[72] Russek SJ. Evolution of GABA a receptor diversity in the human genome. Gene. 1999;227(2):213–22. 10023064 10.1016/s0378-1119(98)00594-0

[73] Pirker S, Schwarzer C, Wieselthaler A, Sieghart W, Sperk G. GABA a receptors: immunocytochemical distribution of 13 subunits in the adult rat brain. Neuroscience. 2000;101(4):815–50. 11113332 10.1016/s0306-4522(00)00442-5

[74] Ding L, Feng H-J, Macdonald RL, Botzolakis EJ, Hu N, Gallagher MJ. The GABA-A receptor alpha 1 subunit mutation A322D associated with autosomal dominant juvenile myoclonic epilepsy reduces the expression and alters the composition of wild type GABA-a receptors. J Biol Chem. 2010;285(34):26390–405. 20551311 10.1074/jbc.M110.142299 PMC2924069

[75] Johannesen K, Marini C, Pfeffer S, Møller RS, Dorn T, Niturad C, et al. Phenotypic spectrum of GABRA1 from generalized epilepsies to severe epileptic encephalopathies. Neurology. 2016;87(11):1140–51. 10.1212/WNL.0000000000003087 27521439

[76] Maljevic S, Krampfl K, Cobilanschi J, Tilgen N, Beyer S, Weber YG, et al. A mutation in the GABA(a) receptor alpha(1)-subunit is associated with absence epilepsy. Ann Neurol. 2006;59(6):983–7. 16718694 10.1002/ana.20874

[77] Ding L, Gallagher MJ. Dynamics of sensorimotor cortex activation during absence and myoclonic seizures in a mouse model of juvenile myoclonic epilepsy. Epilepsia. 2016;57(10):1568–80. 27573707 10.1111/epi.13493 PMC5056152

[78] Kodera H, Ohba C, Kato M, Maeda T, Araki K, Tajima D, et al. De novo GABRA1 mutations in Ohtahara and west syndromes. Epilepsia. 2016;57(4):566–73. 26918889 10.1111/epi.13344

[79] Vorderwülbecke BJ, Kowski AB, Kirschbaum A, Merkle H, Senf P, Janz D, et al. Long-term outcome in adolescent-onset generalized genetic epilepsies. Epilepsia. 2017;58(7):1244–50. 28464258 10.1111/epi.13761

[80] Caraballo R, Silva S, Beltran L, Calvo A, Caballero R. Childhood-only epilepsy with generalized tonic-clonic seizures: a well-defined epileptic syndrome. Epilepsy Res. 2019;153:28–33. 30947078 10.1016/j.eplepsyres.2019.03.017

[81] Camfield P, Camfield C. Idiopathic generalized epilepsy with generalized tonic-clonic seizures (IGE-GTC): a population-based cohort with >20 year follow up for medical and social outcome. Epilepsy Behav. 2010;18(1–2):61–3. 20471324 10.1016/j.yebeh.2010.02.014

[82] Pavlović M, Jović N, Pekmezović T. Antiepileptic drugs withdrawal in patients with idiopathic generalized epilepsy. Seizure. 2011;20(7):520–5. 21493107 10.1016/j.seizure.2011.03.007

[83] Gesche J, Christensen J, Hjalgrim H, Rubboli G, Beier CP. Epidemiology and outcome of idiopathic generalized epilepsy in adults. Eur J Neurol. 2020;27(4):676–84. 31838768 10.1111/ene.14142

[84] Specchio N, Wirrell EC, Scheffer IE, Nabbout R, Riney K, Samia P, et al. International league against epilepsy classification and definition of epilepsy syndromes with onset in childhood: position paper by the ILAE task force on nosology and definitions. Epilepsia. 2022;63(6):1398–442. 35503717 10.1111/epi.17241

[85] Zawar I, Knight EP. Epilepsy with eyelid Myoclonia (Jeavons syndrome). Pediatr Neurol. 2021;121:75–80. 34167046 10.1016/j.pediatrneurol.2020.11.018

[86] Wang XL, Bao JX, Liang S, Tie M, Deng YC, Zhao G, et al. Jeavons syndrome in China. Epilepsy Behav. 2014;32:64–71. 24495864 10.1016/j.yebeh.2013.12.016

[87] Smith KM, Youssef PE, Wirrell EC, Nickels KC, Payne ET, Britton JW, et al. Jeavons syndrome: clinical features and response to treatment. Pediatr Neurol. 2018;86:46–51. 30082241 10.1016/j.pediatrneurol.2018.06.001

[88] Capovilla G, Striano P, Gambardella A, Beccaria F, Hirsch E, Casellato S, et al. Eyelid fluttering, typical EEG pattern, and impaired intellectual function: a homogeneous epileptic condition among the patients presenting with eyelid myoclonia. Epilepsia. 2009;50(6):1536–41. 19490056 10.1111/j.1528-1167.2008.02002.x

[89] Galizia EC, Myers CT, Leu C, de Kovel CG, Afrikanova T, Cordero-Maldonado ML, et al. CHD2 variants are a risk factor for photosensitivity in epilepsy. Brain. 2015;138(Pt 5):1198–207. 25783594 10.1093/brain/awv052 PMC4407192

[90] Wilson MM, Henshall DC, Byrne SM, Brennan GP. CHD2-related CNS pathologies. Int J Mol Sci. 2021;22(2):588. 33435571 10.3390/ijms22020588 PMC7827033

[91] Suls A, Jaehn JA, Kecskés A, Weber Y, Weckhuysen S, Craiu DC, et al. De novo loss-of-function mutations in CHD2 cause a fever-sensitive myoclonic epileptic encephalopathy sharing features with Dravet syndrome. Am J Hum Genet. 2013;93(5):967–75. 24207121 10.1016/j.ajhg.2013.09.017 PMC3824114

[92] Douglas C, Marchion EB, Daud AI, Sullivan DM, Munster PN. Valproic acid alters chromatin structure by regulation of chromatin modulation proteins. Cancer Res. 2005;65(9):3815–22. 15867379 10.1158/0008-5472.CAN-04-2478

[93] Rom A, Melamed L, Gil N, Goldrich MJ, Kadir R, Golan M, et al. Regulation of CHD2 expression by the Chaserr long noncoding RNA gene is essential for viability. Nat Commun. 2019;10(1):5092. 31704914 10.1038/s41467-019-13075-8 PMC6841665

[94] Joshi C, Nickels K, Demarest S, Eltze C, Cross JH, Wirrell E. Results of an international Delphi consensus in epilepsy with myoclonic atonic seizures/ Doose syndrome. Seizure. 2021;85:12–8. 33383403 10.1016/j.seizure.2020.11.017

[95] Ebach K, Joos H, Doose H, Stephani U, Kurlemann G, Fiedler B, et al. SCN1A mutation analysis in myoclonic astatic epilepsy and severe idiopathic generalized epilepsy of infancy with generalized tonic-clonic seizures. Neuropediatrics. 2005;36(3):210–3. 15944908 10.1055/s-2005-865607

[96] Angione K, Eschbach K, Smith G, Joshi C, Demarest S. Genetic testing in a cohort of patients with potential epilepsy with myoclonic-atonic seizures. Epilepsy Res. 2019;150:70–7. 30660939 10.1016/j.eplepsyres.2019.01.008

[97] Hill SF, Meisler MH. Antisense oligonucleotide therapy for neurodevelopmental disorders. Dev Neurosci. 2021;43(3–4):247–52. 34412058 10.1159/000517686 PMC8440367

[98] Miller IO, Sotero de Menezes MA. SCN1A Seizure Disorders: University of Washington: GeneReviews; 1993–2023. 20301494

[99] Bröer S, Gether U. The solute carrier 6 family of transporters. Br J Pharmacol. 2012;167(2):256–78. 22519513 10.1111/j.1476-5381.2012.01975.x PMC3481037

[100] Fattorini G, Melone M, Sánchez-Gómez MV, Arellano RO, Bassi S, Matute C, et al. GAT-1 mediated GABA uptake in rat oligodendrocytes. Glia. 2017;65(3):514–22. 28071826 10.1002/glia.23108

[101] Carvill GL, McMahon JM, Schneider A, Zemel M, Myers CT, Saykally J, et al. Mutations in the GABA transporter SLC6A1 cause epilepsy with myoclonic-atonic seizures. Am J Hum Genet. 2015;96(5):808–15. 25865495 10.1016/j.ajhg.2015.02.016 PMC4570550

[102] Cope DW, Di Giovanni G, Fyson SJ, Orbán G, Errington AC, Lorincz ML, et al. Enhanced tonic GABAA inhibition in typical absence epilepsy. Nat Med. 2009;15(12):1392–8. 19966779 10.1038/nm.2058 PMC2824149

[103] Mermer F, Poliquin S, Zhou S, Wang X, Ding Y, Yin F, et al. Astrocytic GABA transporter 1 deficit in novel SLC6A1 variants mediated epilepsy: connected from protein destabilization to seizures in mice and humans. Neurobiol Dis. 2022;172:105810. 10.1016/j.nbd.2022.105810 PMC9472560 35840120

[104] Schousboe A, Madsen KK, Barker-Haliski ML, White HS. The GABA synapse as a target for antiepileptic drugs: a historical overview focused on GABA transporters. Neurochem Res. 2014;39(10):1980–7. 24627365 10.1007/s11064-014-1263-9

[105] Redon R, Ishikawa S, Fitch KR, Feuk L, Perry GH, Andrews TD, et al. Global variation in copy number in the human genome. Nature. 2006;444(7118):444–54. 17122850 10.1038/nature05329 PMC2669898

[106] Dibbens LM, Mullen S, Helbig I, Mefford HC, Bayly MA, Bellows S, et al. Familial and sporadic 15q13. 3 microdeletions in idiopathic generalized epilepsy: precedent for disorders with complex inheritance. Hum Mol Genet. 2009;18(19):3626–31. 19592580 10.1093/hmg/ddp311 PMC3465696

[107] Mefford HC, Muhle H, Ostertag P, von Spiczak S, Buysse K, Baker C, et al. Genome-wide copy number variation in epilepsy: novel susceptibility loci in idiopathic generalized and focal epilepsies. PLoS Genet. 2010;6(5):e1000962. 10.1371/journal.pgen.1000962 PMC2873910 20502679

[108] Moreau C, Tremblay F, Wolking S, Girard A, Laprise C, Hamdan FF, et al. Assessment of burden and segregation profiles of CNVs in patients with epilepsy. Ann Clin Transl Neurol. 2022;9(7):1050–8. 35678011 10.1002/acn3.51598 PMC9268881

[109] Kirov A, Dimova P, Todorova A, Mefford H, Todorov T, Saraylieva G, et al. 15q13. 3 microdeletions in a prospectively recruited cohort of patients with idiopathic generalized epilepsy in Bulgaria. Epilepsy Res. 2013;104(3):241–5. 23352738 10.1016/j.eplepsyres.2012.10.013

[110] Fejgin K, Nielsen J, Birknow MR, Bastlund JF, Nielsen V, Lauridsen JB, et al. A mouse model that recapitulates cardinal features of the 15q13. 3 microdeletion syndrome including schizophrenia-and epilepsy-related alterations. Biol Psychiatry. 2014;76(2):128–37. 24090792 10.1016/j.biopsych.2013.08.014

[111] Thakran S, Guin D, Singh P, Singh P, Kukal S, Rawat C, et al. Genetic landscape of common epilepsies: advancing towards precision in treatment. Int J Mol Sci. 2020;21(20):7784. 10.3390/ijms21207784 PMC7589654 33096746

[112] Damiano JA, Mullen SA, Hildebrand MS, Bellows ST, Lawrence KM, Arsov T, et al. Evaluation of multiple putative risk alleles within the 15q13. 3 region for genetic generalized epilepsy. Epilepsy Res. 2015;117:70–3. 26421493 10.1016/j.eplepsyres.2015.09.007

[113] Coppola A, Bagnasco I, Traverso M, Brusco A, Di Gregorio E, Del Gaudio L, et al. Different electroclinical picture of generalized epilepsy in two families with 15q13. 3 microdeletion. Epilepsia. 2013;54(5):e69–73. 23448223 10.1111/epi.12130

[114] Heinzen EL, Radtke RA, Urban TJ, Cavalleri GL, Depondt C, Need AC, et al. Rare deletions at 16p13. 11 predispose to a diverse spectrum of sporadic epilepsy syndromes. Am J Hum Genet. 2010;86(5):707–18. 20398883 10.1016/j.ajhg.2010.03.018 PMC2869004

[115] Jiang Y, Zhang Y, Zhang P, Sang T, Zhang F, Ji T, et al. NIPA2 located in 15q11. 2 is mutated in patients with childhood absence epilepsy. Hum Genet. 2012;131(7):1217–24. 22367439 10.1007/s00439-012-1149-3

[116] Møller R, Hjalgrim H. Submicroscopic chromosomal changes predispose to generalised epilepsy. Ugeskr Laeger. 2011;173(16–17):1201–4. 21501562

[117] Mullen SA, Carvill GL, Bellows S, Bayly MA, Berkovic SF, Dibbens LM, et al. Copy number variants are frequent in genetic generalized epilepsy with intellectual disability. Neurology. 2013;81(17):1507–14. 24068782 10.1212/WNL.0b013e3182a95829 PMC3888172

[118] Muhle H, Mefford HC, Obermeier T, Von Spiczak S, Eichler EE, Stephani U, et al. Absence seizures with intellectual disability as a phenotype of the 15q13. 3 microdeletion syndrome. Epilepsia. 2011;52(12):e194–8. 22050399 10.1111/j.1528-1167.2011.03301.x PMC3270691

[119] Jabbari K, Bobbili DR, Lal D, Reinthaler EM, Schubert J, Wolking S, et al. Rare gene deletions in genetic generalized and Rolandic epilepsies. PLoS One. 2018;13(8):e0202022. 30148849 10.1371/journal.pone.0202022 PMC6110470

[120] Bureau M, Tassinari CA. Epilepsy with myoclonic absences. Brain and Development. 2005;27(3):178–84. 15737698 10.1016/j.braindev.2004.01.008

[121] Zanzmera P, Menon RN, Karkare K, Soni H, Jagtap S, Radhakrishnan A. Epilepsy with myoclonic absences: Electroclinical characteristics in a distinctive pediatric epilepsy phenotype. Epilepsy Behav. 2016;64(Pt A):242–7. 27770719 10.1016/j.yebeh.2016.08.023

[122] Mangano S, Fontana A, Spitaleri C, Mangano GR, Montalto M, Zara F, et al. Benign myoclonic epilepsy in infancy followed by childhood absence epilepsy. Seizure. 2011;20(9):727–30. 21752671 10.1016/j.seizure.2011.06.008

[123] Jiang Y, Zhou X. Psychomotor development and seizure features in idiopathic myoclonic epilepsy in infancy. Medicine (Baltimore). 2022;101(38):e30512. 10.1097/MD.0000000000030512 PMC9509150 36197249

[124] Sokka A, Olsen P, Kirjavainen J, Harju M, Keski-Nisula L, Räisänen S, et al. Etiology, syndrome diagnosis, and cognition in childhood-onset epilepsy: a population-based study. Epilepsia Open. 2017;2(1):76–83. 29750215 10.1002/epi4.12036 PMC5939454

[125] Zhang J, Chen J, Zeng Q, Zhang L, Tian X, Yang X, et al. Infantile epilepsy with multifocal myoclonus caused by TBC1D24 mutations. Seizure. 2019;69:228–34. 31112829 10.1016/j.seizure.2019.05.010

[126] Koko M, Motelow JE, Stanley KE, Bobbili DR, Dhindsa RS, May P. Association of ultra-rare coding variants with genetic generalized epilepsy: a case-control whole exome sequencing study. Epilepsia. 2022;63(3):723–35. 35032048 10.1111/epi.17166 PMC8891088

